# A recirculating device of cooling water powered by solar energy for the laboratory

**DOI:** 10.1038/s41598-024-66215-6

**Published:** 2024-07-06

**Authors:** Jiahui He, Wenhao Deng, Maochun Zhu, Gearóid M. Ó. Máille, Zihang Wu, Longsheng Wang, Yongge Wei

**Affiliations:** 1 School of Materials and Chemical Engineering, Hubei University of Technology, Wuhan, 430068 P.R. China; 2 Department of Chemistry, Trinity College Dublin, Dublin, D02 PN40 Ireland; 3 School of Chemistry, Tsinghua University, Beijing, 10084 P.R. China

**Keywords:** Chemical education, Organic chemistry

## Abstract

Aimed at energy conservation and water saving for the lab, we have designed and constructed one kind of lab-scale small recirculating device of cooling water utilizing a water recirculator coupled to a solar energy system via a self-made multifunctional voltage regulator, which is equipped with an active heat radiator and powered by a solar energy system. It can provide cooling water for 1–3 sets of ordinary refluxing setups in series without additional consumption of water and electricity. The temperature difference between the water in the bucket and the environment is less than 4 °C for eight common solvents in single refluxing set-up or three combined refluxing setups with different solvents in series. In the performance assessment experiments for the refluxing of eight common solvents with different boiling point, the largest solvent loss is less than 6% if the condenser is open to the air in the refluxing time of 8 h, but none obvious solvent loss are found if the condensers were equipped with an oil bubbler. Control experiments indicates that the preparation of bromoethane/ethyl acetate/propyl hexanoate using our water recirculator can achieve almost unanimous yields in relative to those reactions using tap water as cooling water.

## Introduction

Consumption of fossil fuels, such as coal, oil, and natural gas, has occupied a great proportion currently. As one-off and non-renewable energy, their limited reserves will result in inevitable energy crisis in the future. Moreover, combustion of fossil fuels had produced CO_2_, SO_2_, nitrous oxides and particulate matter 2.5(PM2.5), which had brought out some environmental problems, such as global warming and acid rain. Therefore, it is necessary to find clean and renewable energy sources to replace fossil fuels^1^. Solar energy is permanent, universal, and inexhaustible as a clean and renewable energy. The promotion and popularization of solar power installations can not only reduce the consumption of electricity and fossil fuels, but also lower the emissions of pollutants such as CO_2_, SO_2_, nitrous oxides and PM2.5^2^.

Water is the source of life. Seventy percent of earth surface is covered with water. It seems that water is “abundant” in our earth. However, fresh water occupied only ca. 2.53% of total water. Most of fresh water (68.69%) are stored in the glaciers of high mountains, South poles and North poles^3^. Moreover, the total amount of fresh water continues to decrease due to the global warming. Therefore, water-saving is one very urgent issue for us to realize the sustainable development. To achieve the goal of energy conservation and water-saving, household energy-saving behavior play an important role^4^, the invention of novel water-saving devices and their promotions in our daily life is very essential besides the popularization of water-saving ideas^5,6^.

For many chemical labs, cooling is still necessary in many experimental operations, such as refluxing, distillation and rotary evaporation^7^. Currently, the available cooling approaches in the lab are circulating cryostat or tap water. Circulating cryostats have been used for rotary evaporator or other special equipment due to their good cooling ability^8^. However, their high costs restrict their extensive applications in the lab. Moreover, they are relatively high energy consumption, for example, the power consumption of a Buchi Recirculating Chiller F305 (800W in full power) is about 19.2 kW h or 9.6 kW h in one day if it works at the full power or half full power. Tap water has been adopted as cooling means in most chemical labs because it is readily available and cost-effective compared to circulating cryostats. However, there are two obvious disadvantages to use tap water as cooling means. Firstly, water is extremely wasteful if the used tap water goes down the drain directly: one faucet can drain away fresh water about 720–2880 L/day with the flow rate of 0.5–2 L/min. Secondly, it will possibly lead to flooding in the lab if water pipe is broken or water faucet is unclosed^9^. Recently, some improved air condensers or condenser kits, for example, Waterless Condenser (Asynt Corp)^10^ have been emerged and available commercially. However, their high price was one disadvantageous factor for their large-scale applications in the laboratory. Moreover, both tap water and circulating cryostats are not the best choice for most condensation from the standpoint of the energy efficiency and efficient water resource ultilization, the former will result in the waste of fresh water inevitably, the circulating cryostat with strong cooling ability is designed for rotary evaporator with high cooling requirement but is superfluous for most distillation or refluxing operation that need condensation. The key issue is how to expel those heat generated in condensation process into the environment as soon as possible. Therefore, it is still high-priority for the lab to seek an inexpensive and efficient cooling device that addresses the drawbacks of using tap water or circulating cryostats.

Up to now, some devices had been developed to improve the supply of cooling water within the lab. These improvements majorly focused on two strategies: the first one is to customize a special air condenser or a condenser with a static liquid jacket. For example, Grist et al. have reported a novel condenser utilizing a Vigreux column surrounded by a water jacket that is encased in a finned aluminium tube^11^. Baum et al. had designed a static fluid condensers with a glycol jacket^12^. Lunelli et al. had invented one kind of cone-shape air condenser suitable for the small-scale or semi-microscale reflux^13^. However, the passive cooling nature of such condensers renders them only suitable for the long time reflux of the common solvents with low boiling point or the short time reflux of these solvents with high boiling point. Moreover, numerous condensers existing in chemical labs will become useless if such custom-made condensers are applied extensively. The second one is to build a circulating device of cooling water. For example, Fleming et al. have reported a self-circulating system using ice-bath to cool the cooling water, which is suitable for a small scale reflux^14^. Ding had designed a water recirculation system using booster pump, water storage tank and water channel, which can be mounted under the experimental table^15^. Pelletier et al. recently reported a low-cost recirculation system mounted on the experimental table^16^. Although these devices without the active cooling device can alleviate the waste of water to some extent, they are still confronted with the issue of prolonged heat accumulation for the long time reflux or high-intensity reflux, which will lead to the raise of the water temperature and bring down their cooling efficiency. Moreover, they cannot work without electricity. Consequently, it would greatly benefit laboratories to have access to affordable and energy-efficient recirculating cooling devices that incorporate an active cooling device, offering convenience and sustainability.

Aimed to replace tap water as cooling water in the lab, we had designed one kind of separate combined water recirculator for the lab in 2017^17^ and one kind of integrated water recirculator for the lab in 2019^18^. Given the increasing electricity costs and the tendency of global warming, energy conservation had become the daily life for everyone to strike the target of carbon neutrality. Solar energy as one kind of clean and renewable energy^19^ is a promising substitution of fossil energy for greenhouse gases mitigation^20^. By combing the water recirculator and the solar energy system, we had designed and constructed one kind of recirculating device of cooling water with active cooling device powered by solar energy system, which consisted of a submerged pump, a water tank and a heat exchanger, one solar panel, one lead-acid storage battery and one self-made voltage regulator. This device can provide cooling water for the lab without the consumption of electricity and tap water. This device not only is inexpensive, but also can reduce the consumption of electricity and water to a great extent. Herein, we report the design, construction and performance assessment of the solar energy-driven water recirculating cooling device.

## Experimental material and methods

All chemicals were purchased from Sinopharm Chemical Reagent Co., Ltd or Hewons Biochemical Technology Co., Ltd. All solvents were used as received without further purification. Deionized water was obtained from AWL-4001-B (Auapro International Company LLC).

For all control experiments, the 250 mL round flask filled with 100 mL solvent was refluxed using oil bath for eight hours. The flask was equipped with identical 300 mm Allihn condensers with an oil bubbler (Condition A) or open to the air (Condition B). The working voltage of pump and the electric fan on the heat exchange radiator is 5 V (condition 1), 9 V (condition 2) or 12 V (condition 3). Solvent loss rate can be calculated according to Eq. (1). The common mercury thermometer with the range of 0–200 °C was used to measure the temperature of reflux or distillation operation, and the electronic temperature sensor was used to measure the water temperature of the water reservoir.

1 $$\begin{aligned} {{Solvent\;loss(\%)} = (V_{start} - V_{remain})\div V_{start}\times 100\%} \end{aligned}$$

Bromoethane (CH_3_CH_2_Br) was prepared according to the following procedure^21^: 14.5 mL H_2_SO_4_ (72%) and 6.5 g NaBr were added to a round flask (50 mL) in the ice-water bath, then followed by 5 .0 mL alcohol (95%). The mixture was heated using a simplified distilling apparatus. The mixture was refluxed using low voltage (there should be not distillate in the first 0.5 h to 2 h according to the quantity of reactants). Then, the crude bromoethane were distilled by raising the temperature and dried by concentrated H_2_SO_4_ (98%), the dry bromoethane was further refined by distillation to collect the fraction between 37–40 °C.

Ethyl acetate (CH_3_CO_2_C_2_H_5_) was prepared according to the following procedure:^21^ 1.5 mL H_2_SO_4_ (98%) and 1.5 mL CH_3_CO_2_H was placed in a three-necked flask (50 mL) and heated to 120 °C under refluxing using a set of dropwise Add. -fractionating setup. In the period of about 45min, the mixture of 7.8 mL alcohol and 7.2 mL CH_3_CO_2_H was dripped slowly to the mixture of CH_3_CO_2_H and H_2_SO_4_ to maintain the reaction temperature in the range of 120–125 °C. The mixture was refluxed for another 5 minutes until without distillate. The crude ethyl acetate was washed by saturated sodium bicarbonate solution and successively by brine, then dried by MgSO_4_. The dry ethyl acetate was refined by distillation to collect the fraction between 74–80 °C.

Propyl hexanoate was prepared according to the following procedure: 12.6 mL hexanoic acid (11.6 g, 0.10 mol), 15.0 mL n-propanol (12.0 g, 0.20 mol), 15.0 mL benzene and 0.50 g phosphotungstic acid (0.17 mmol) were added to a single-necked round flask (100 mL) successively, the mixture was refluxed until to yield about 1.8 mL water through the equipped water separator. The obtained mixture was cooled to room temperature, and the solvent was removed by rotary evaporation. The remained organic phase was washed successively by 15 .0 mL water, 10.0 mL saturated sodium bicarbonate solution and 10.0 mL saturated saline solution, then dried by MgSO_4_. The propyl hexanoate was distilled under reduced pressure.

## Results

**Figure 1:**
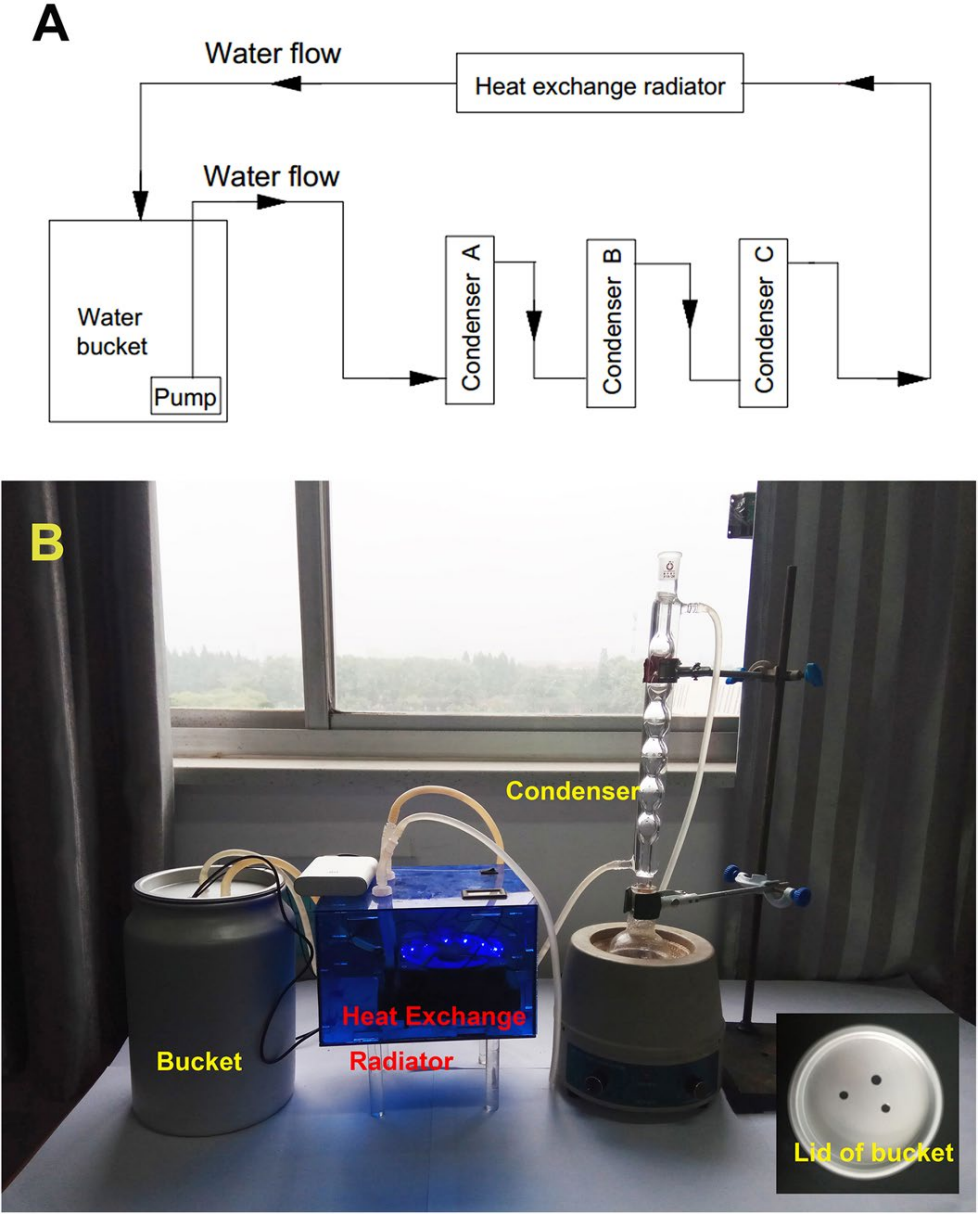
(**A**) The flow chart of the designed water recirculator, the direction of the arrow in the chart represents the flow direction of the water. (**B**) The photograph of the water recirculator.

The device includes a power system and a water recirculator. The former provides power for the device, and the later supplies cooling water for condensers and dissipate the accumulated heat into the environment. They are linked through a self-made multifunctional voltage regulator (see Fig. 1), which can adjust the input voltage for the water recirculator and electric fan on the heat exchange radiator.

**Figure 2:**
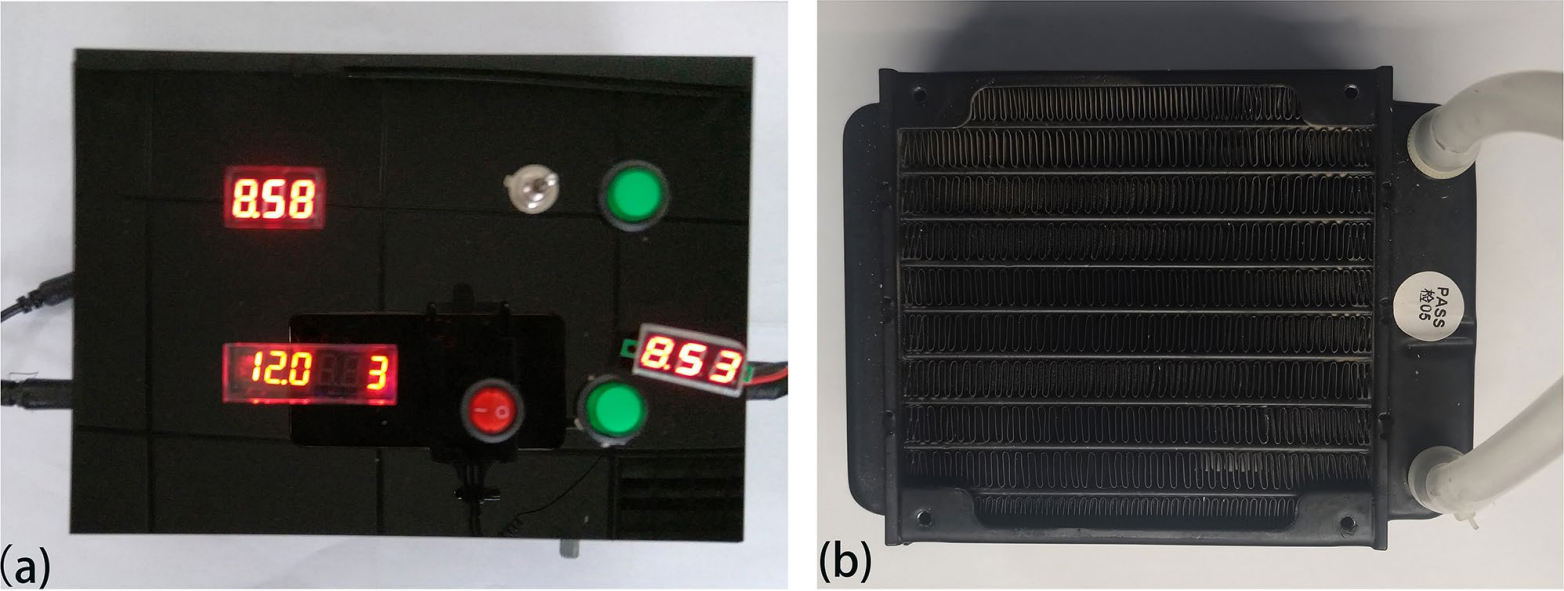
(**a**) Photograph of the multifunctional voltage regulator; (**b**) Photograph of the heat exchanger.

The key of this device to save water is using an economic and practical water recirculator instead of tap water. Inspired by the water filtration system of aquarium, we had designed and constructed a water recirculator suitable for the lab. The flow chart and photograph of the water recirculator were shown in Fig. 1. It is made up of a closed aluminium bucket, a submerged mini-pump, several connecting pipes and a heat exchange radiator (Fig. 2b), one commercial water cooling radiator with the size of 120 × 120 mm was used as the heat exchange radiator. Deionized water (tiny biocide was added to avoid biofilms and algae) is stored in an aluminium bucket (5 L) with a lid , the aluminium bucket was chosen as water reservior because aluminium has better heat dissipation capacity and lower costs compared to other materials (Table S3 and Fig. S9). Three holes are drilled on the lid, two holes are connected with the circulating water outlet and inlet, the third one is used to pass through the pump wire and electric thermometer. One submerged mini-pump is mounted on the bottom of the aluminium bucket to connect the water outlet via a pipe. The water outlet is connected to a condenser, then linked with a heat exchange radiator, which is linked with the water inlet of the bucket to form a closed water circuit system. The water recirculator renders the coolant all along running in a closed loop and can economized lots of water instead of tap water. Power system consists of a solar panel, a solar charge controller and a lead-acid storage battery. The solar panel is 18 V/100 W; the lead-acid storage battery is 12 V/100 AH (The details to calculate the power of solar panel and the capacity of storage battery was shown in the Electronic Supporting Information); and the solar charging controller is of 12 V/24 V/30 A. (Note: Insulating the battery terminals can avoid accidental short circuit).

The nominal voltage of the submerged pump and electric-fan of radiator for the water recirculator is 12 V, they can work in a voltage range of 5–12 V. Therefore, a DC supply between 5–12 V is necessary for the water recirculator. However, the output voltage of solar panel is 18 V, the output voltage range of lead-acid storage battery is 10.8–13.5 V, the output voltage of solar charge controller and power bank is 5 V. Therefore, a device with the ability to adjust the voltage of different sources into the range of 5–12 V is necessary to drive the water recirculator. Therefore, we designed and made a multifunctional voltage regulator (see Fig. 2a), the circuit diagram and the details can be seen in the Electronic Supporting Information.

Before connecting the power system and water recirculator, we tested the output voltage of our voltage regulator upon linking to different electric sources (see Table 1). These results indicate that our voltage regulator can step down the input voltage of solar panel (15–20 V) and the lead-acid battery (13.1–10.6 V) to a range of 8.5–13.4 V using input port I; input port II of the voltage regulator can raise the output voltage to 5 V, 9 V, and 12 V, respectively, when linked with 5 V DC or power bank. Therefore, the self-made voltage regulator can realize the function of voltage transformation in different situations and satisfy the voltage demand of the water recirculator.

**Table 1:** The output voltage through multifunctional voltage regulator.

Input port	Input type	Input voltage (/V)	Output voltage (/V)
I	Solar panel	15–20	8.7–13.4
I	Storage battery	13.1–13.6	8.7–12.5
I	12V DC	12	8.7
II	Power bank	5.0	3.74, 9.04, 11.9
II	5V DC	5.0	4.67, 9.07, 11.6

**Figure 3:**
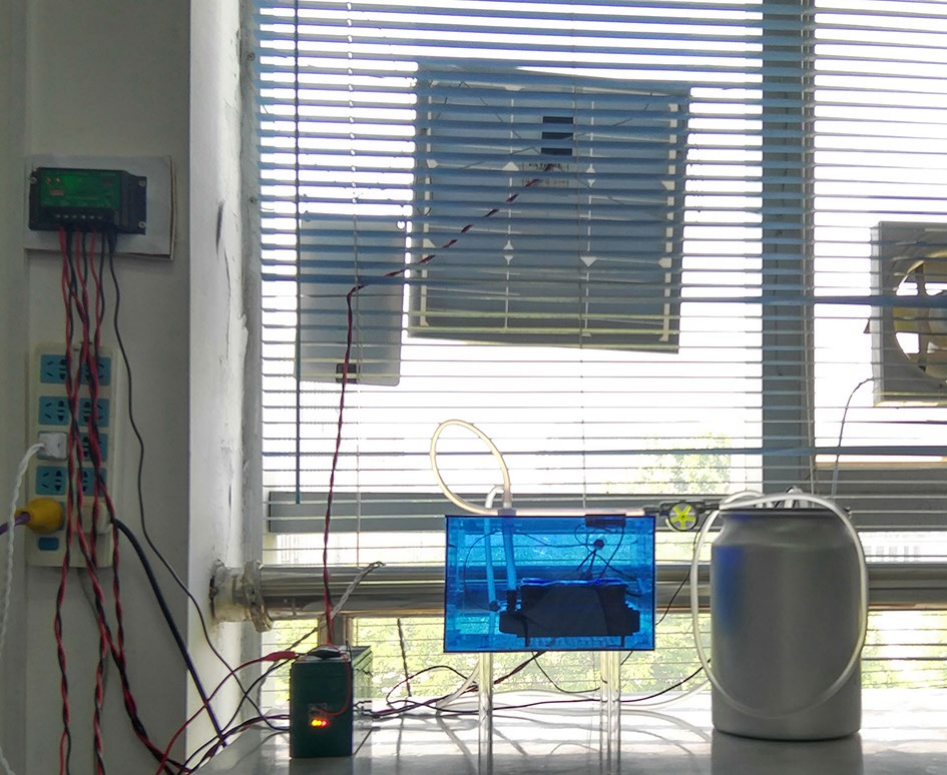
Photograph of the combined device including energy system and water recirculator. (Note: The water recirculator can be placed on the experimental table or fume cupboard using a long electric wire).

The electricity generated by solar panel was stored in the lead-acid storage battery in the daytime. The water recirculator was powered by the lead-acid storage battery. The water recirculator can also work using a DC adaptor (5–12 V). The multifunctional voltage regulator can adapt different electric sources such as a lead-acid battery (10–14 V), a power bank (5 V) and a DC adapter (5 V), and regulate different input voltages into the range of 5–12 V to ensure the smooth running of the whole system. The pump drives the water to flow through the condenser and brings heat to the heat exchange radiator. Most of heat are expelled into the environment here, and the water flows back into the aluminium bucket to form a water circuit system, in which another part of heat are exchanged with the environment via the bucket wall. The combined device is shown in Fig. 3, the working video of the whole device can be found in the Electronic Supporting Information.

**Figure 4:**
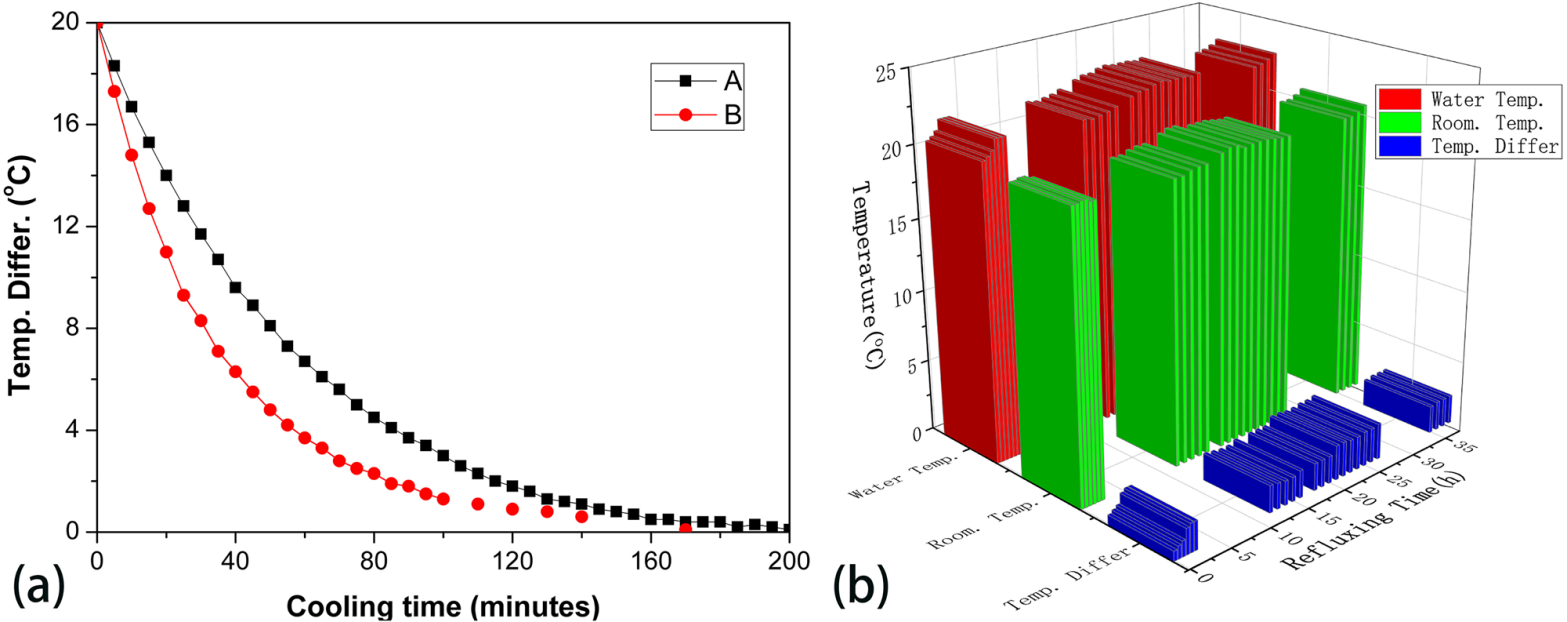
(**a**) The natural cooling cure of the bucket (A) and the cooling curve of the bucket equipped with one heat exchanger (B); (**b**) The water temperature, room temperature and temperature difference of 100 mL water reluxed at 120 °C oil bath for 38 h using the water recirculator.

Before to evaluate the cooling performance of this device, we firstly to inspect its heat dissipation ability. Using 4.5 L hot water filled in the Al bucket with the temperature above about 20 °C than room temperature (R. T.), the natural cooling curve of the Al bucket and the cooling curve of the Al bucket equipped with one heat exchange radiator were obtained by measuring the water temperature at the interval of half an hour, respectively (As shown in Fig. 4a). The results indicate that the larger temperature difference of water and R.T., the better heat dissipation ability in both experiments, but our device demonstrate more excellent heat dissipation ability than the bucket. The water temperature of our device can bring down about 15 °C in fifty minutes, while the bucket without the heat exchanger can only lower about 8 °C in the same time. Our device also exhibit excellent heat dissipation ability even if the temperature difference is less than 2 °C. In order to assess the cooling effect of our device, eight common solvents were chosen to be refluxed for 8 hours using different refluxing condition: a refluxing setup of 100 mL solvent in 250 mL round flask equipped with an Allihn type condenser (30 cm) were heated by oil bath, the condenser is open to the air (condition a), or the condenser is equipped with an oil bubbler (condition b). The working voltage of pump and the electric fan on the heat exchange radiator is 5 V (condition 1), 9 V (condition 2) or 12 V (condition 3). Solvent loss and the temperature difference of those solvents in different refluxing conditions are summarized in Table 2.

**Table 2:** Performance assessment of the equipment using single reflux set-ups with different common solvents.

Solvent	Water	Ethanol	Acetonitrile	Acetone	THF	Hexane	DCM	Acetic acid
B. P. (°C)	100.0	78.3	81.6	56.0	65.0	68.7	40.0	118
Oil bath (°C)	120	100	100	80	80	90	80	140
Reflux time (h)	8	8	8	8	8	8	8	8
Temp. differ.(°C)^1,a^	3.6	2.9	2.1	2.1	2.2	2.7	2.5	4.2
Temp. differ.(°Cs)^2,a^	2.6	1.6	1.3	1.2	1.6	0.9	1.2	2.9
Temp. differ.(°C)^3,a^	2.6	1.6	1.9	0.9	1.8	1.3	1.2	3.1
Solvent loss ^1,a^	3%	5%	5%	6%	5%	5%	6%	4%
Solvent loss ^2,a^	2%	3%	2%	5%	4%	3%	4%	2%
Solvent loss ^3,a^	2%	3%	2%	4%	4%	3%	4%	1%
Solvent loss ^1,b^	0%	0%	0%	0%	0%	0%	0%	0%
Solvent loss ^2,b^	0%	0%	0%	0%	0%	0%	0%	0%

^1^ working voltage of pump and electric fan is 5 V; ^2^ working voltage of pump and electric fan is 9 V; ^3^ working voltage of pump and electric fan is 12 V; ^a^ the condenser is open in the air; ^b^ the condenser is equipped with an oil bubbler. Temp. Differ. is calculated using the temperature of water in the bucket (T_water_) minus the room temperature (T_r.t._). Solvent loss (%) = (V_start_ – V_remain_)/V_start_ × 100%.

It can be seen that all solvents have not any obvious volume loss in the refluxing time of 8h if the condenser is equipped with an oil bubbler (see Table 2). The largest solvent loss for all tested solvents is no more than 6% if the condenser is open to the air. The temperature difference between the water in the bucket and the ambient temperature depend on the boiling point and the vaporization heat of solvent: the higher the boiling point and the vaporization heat of solvent, the higher the temperature difference. If the working voltage of pump and electric fan on the heat exchange radiator are raised to 9 V or 12 V, the solvent loss and temperature difference can be further lowered owing to the faster speed of electric fan and the faster flow velocity of water compared to the working voltage of 5 V.

Considering some experiments possibly need a extended reaction time, to investigate its cooling performance under long-time refluxing, 100 mL water in a 250 mL single-necked ground-flask was refluxed for 38 h under 120 °C oil bath, the water temperature, room temperature and their temperature difference were recorded at the interval of one hour (As shown in Fig. 4b). This result indicates that the temperature difference reached nearly a balance after two hours refluxing, and its value range from 1.8 to 2.5 °C during the reflux of 38 h. This experiment account for that our device is qualified to provide cooling water for the long time refluxing. To further validate the cooling ability of our device, three serial refluxing setups were used to evaluate the cooling effect of our water recirculator. These condensers of three refluxing setups of 100 mL water, 100 mL ethanol and 100 mL DCM are connected in series to one water recirculator, or vice versa. There is also not obvious solvent loss in the 8 h reflux if their condensers are equipped with oil bubblers (Table 3). Without oil bubbler, the solvent loss is no more than 6%, and the temperature difference is less than 4 °C. Therefore, our recirculator not only has a good cooling ability for the reflux of common solvents, but also can be qualified for the reflux of three serial refluxing set-ups of common solvents.

**Table 3:** Performance assessment of the equipment using three refluxing set-ups.

Solvents	Water	Ethanol	DCM	DCM	Ethanol	Water
Linking order	1	2	3	1	2	3
Boiling point (°C)	100.0	78.3	40.0	40.0	78.3	100.0
Heating temp.(°C)	126	92	80	80	92	126
Reflux time (h)	10	10	10	10	10	10
Solvent loss ^1,a^	3%	5%	6%	5%	4%	4%
Solvent loss ^1,b^	0	0	0	0	0	0

^1^ working voltage of pump and electric fan is 9 V; ^a^ the condenser is open in the air; ^b^ the condenser is equipped with an oil bubbler. Temp. Differ. are calculated using the temperature of water in the bucket (T_water_) minus the room temperature (T_r.t._). Solvent loss(%) = (V_start_ – V_remain_)/V_start_ × 100%.

Two teaching organic experiments, including the preparation of bromoethane and the preparation of ethyl acetate in our school, were used to evaluate the practical effects of our water recirculator. The yields of bromoethane/ethyl acetate in these experiments using the water recirculator (A) or running water (B) as cooling water were listed in Table 4. Additionally, propyl hexanoate were also prepared using the water recirculator or tap water as cooling water, the yields of propyl hexanoate were also listed in Table 4. It’s easily found that the average yields of bromoethane /ethyl acetate/propyl hexanoate using water recirculator are 44.4%, 44.5%, 75.7%, respectively, which are close to or slightly lower than (44.5%, 47.9%, 77.5%) of those experiments using running water. It’s indicated that our water recirculator can replace running water as cooling water in the daily teaching of organic experiments or organic synthesis that needs the reflux or distillation operation.

**Table 4:** The yield of Bromoethane/ethyl acetate/ propyl hexanoate.

	Bromoethane (%)	Ethyl acetate (%)	Propyl hexanoate (%)
A	48.0	45.9	78.6
A	45.1	39.9	70.4
A	40.0	47.6	78.0
B	48.7	47.6	79.9
B	41.1	49.3	73.5
B	43.6	46.7	79.2

A: Water recirculator; B: Tap water.

Instead of one faucet with the water flow rate of 0.5–2 L/min, our device can save water about 720–2880 L/day for the lab. Powered by the solar energy system, water recirculator can uninterruptedly provide cooling water for the lab, it can work for three days (depending on the capacity of storage battery) in cloudy day or rainy weather without sunlight. There is no doubt that the water recirculator can be driven by mains electricity or power bank without solar energy system. The power consumption of our water recirculator is 0.24 kW h/day. By roughly estimation, it can economize electricity about 18.96 kW h or 9.36 kW h in one day instead of a circulating cryostats (800 W) if the circulating cryostats worked at the full power or half full power. The extensive application of such water recirculator will conserve fresh water and electricity for the lab to a great extent.

## Conclusions

Energy conservation and water saving have attracted more and more attentions for meeting the requirement of sustainability in the chemical lab. In this work, we have designed and constructed a practical water recirculator powered by solar energy system. It can be an eco-friendly alternative to replace tap water or circulating cryostats to provide cooling water for the lab. This device, without the consumption of water, has the advantages of cost-effective, lower energy consumption, simple structure, and easy maintenance. It can reflux one to three sets of refluxing setups with different solvents. Its extensive application in chemical laboratories will save lots of water and electricity. By comparing the yields of bromoethane/ethyl acetate/propyl hexanoate, those experiments using the water circulator as cooling methods had achieved almost unanimous yields in relative to those using tap water, indicated that the water circulator can replace tap water as coolant in the daily organic experimental teaching or organic synthesis.

### Supplementary Information

Supplementary Legends.

Supplementary Information 1.

Supplementary Video 1.

Supplementary Video 2.

## Data Availability

Data is provided within the manuscript or supplementary information files. The online version contains supplementary material is available at 10.1038/s41598-024-66215-6. Correspondence and requests for materials should be addressed to L.W. or Y.W.
