# Supplementary material for: A recirculating device of cooling water powered by solar energy for the laboratory
Source: Sci Rep. 2024 Jul 6;14:15572. doi: 10.1038/s41598-024-66215-6 (PMC11227590; doi:10.1038/s41598-024-66215-6)
Supplement: Supplementary file 1 — Supplementary Legends. [file 41598_2024_66215_MOESM1_ESM.docx]

**Legend for Electronic Supplementary Materials**

1. Supporting Information

PART I DETAILS OF HARDWARE

Building Blocks of Energy System

Building Blocks of Self-made Multifunctional Voltage Regulator

PART II THE PRICE OF THOSE BUILDING BLOCKS

Part III THEORETICAL CALCULATION

1. Work Video I

The water recirculator was driven by power bank.

1. Work video II

The water recirculator was driven by the solar system.
