# Supplementary material for: A recirculating device of cooling water powered by solar energy for the laboratory
Source: Sci Rep. 2024 Jul 6;14:15572. doi: 10.1038/s41598-024-66215-6 (PMC11227590; doi:10.1038/s41598-024-66215-6)
Supplement: Supplementary file 2 — Supplementary Information 1. [file 41598_2024_66215_MOESM2_ESM.docx]

Supporting Information

A Recirculating Device of Cooling Water Powered by Solar Energy for the Laboratory

**Jiahui He^1,ǂ^, Wenhao Deng^1,ǂ^, Maochun Zhu^1,ǂ^, Gearóid M. Ó Máille ^2^, Zihang Wu^1^, Longsheng Wang^1,^* and Yongge Wei^3,^***

*^1^* *School of Materials and Chemical Engineering, Hubei University of Technology, Hubei Wuhan, 430068, P.R. China.*

*^2^* *Department of Chemistry, Trinity College Dublin, D02 PN40, Ireland.*

*^3^* *School of Chemistry, Tsinghua University, Beijing 10084, P.R China*

^≠^These authors contributed equally to this work.

PART I DETAILS OF HARDWARE

Building Blocks of Energy System

Building Blocks of Self-made Multifunctional Voltage Regulator

PART II THE PRICE OF THOSE BUILDING BLOCKS

Part III THEORETICAL CALCULATION

PART I DETAILS OF HARDWARE

Building Blocks of Energy System

The schematic and photograph of multifunctional voltage regulator are shown in Figure 1 in the paper, and here we provide the details of building blocks.

**Solar panel:**

The single crystalline silicon solar panel, which has a solar cell efficiency of 16.5%, was made in China and purchased from Taobao. The solar panel (working voltage of 18V, open circuit voltage of 21.6V, working current of 1.13A and the power of 20W) was selected for the demonstration device. Another solar panel (the working voltage of 17.5V, open circuit voltage of 21.5V, working current of 5.71A and the power of 100W) can drive the work device for three days. They can work in the temperature range of -40^o^C ~ 80^o^C. The single crystalline silicon panel is most expensive investment in this device, but it has the longest working life of 15-20 years.


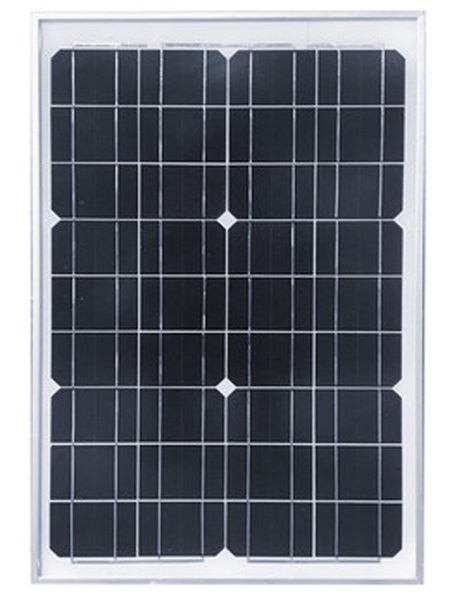


Figure S1. The photograph of solar panel.

**Solar charge controller:**

Solar charge controller (Guanghe, BW-KZQ-10AK) is a common positive PWM charge controller with LCD display. It adopts the most advanced digital technique. The multiple load control modes enable it can be widely used in solar home system, traffic signal, solar street light, solar garden lamp and so on. The controller has the self-recognition function on the input voltage of 12V/24V with the nominal working current of 10A. It has one output port of 12V and one output port of 5V. The 12V output port can drive the device directly in a higher cooling performance. The output port of 5V can charge for most power bank or drive the device in a lower cooling performance.


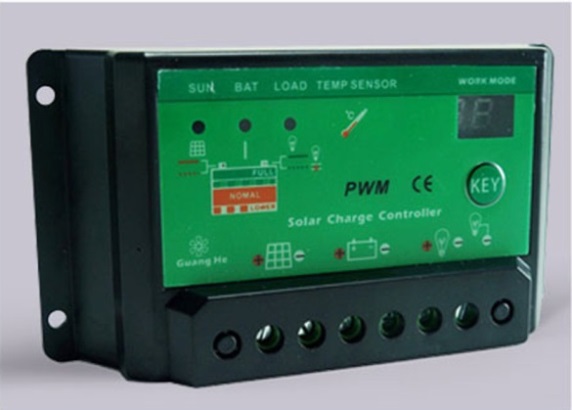


Figure S2 The photograph of solar charge controller.

**Storage battery**

Considering the higher working voltage and lower price, the lead-acid battery of 12V/12Ah designed for solar system is selected as the storage battery for he demonstrating device. And the lead-acid battery of 12V/100Ah is selected for purpose to work for a week without sunshine. This kind battery using gas silicon dioxide, small granule degree, bigger than surface area, has a high thermal capacity. It not only can reduce the risk of thermal out of control and drying hard, but also can be used in bad environment. The maximum design life of the battery is up to 5-8 years. It has the virtues of long time discharge, long storage time, and good deep discharge resilience performance. Therefore, it can meet the demand of this device.


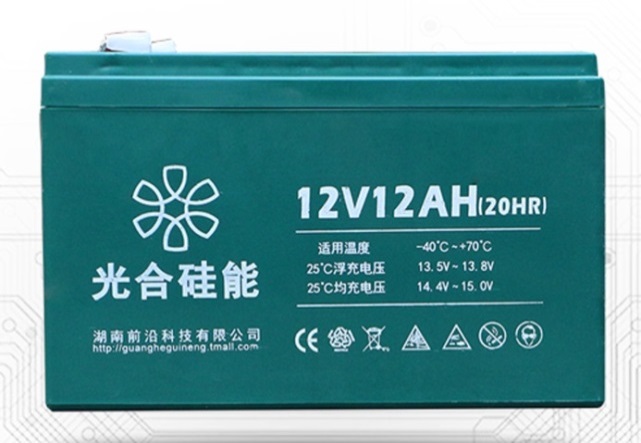


Figure S3. The photograph of silicon acid-lead storage battery

Building Blocks of the Recirculator

**Submerged Pump (Jiatong, JT160)**

The submerged pump (DC) is selected the type with the range of nominal voltage at 6 ~ 12V, the pump head of 2.2m and the rate of flow 240L/h, the range of working current is 0.15 ~ 0.35A. It is necessary that the pump have a wide range of working voltage and enough pump head to meet with the demand to pump the cooled water for the system. The wide working voltage render it suitable for different input voltage of different source, such as solar panel, storage battery, power bank, DC supply, and so on.


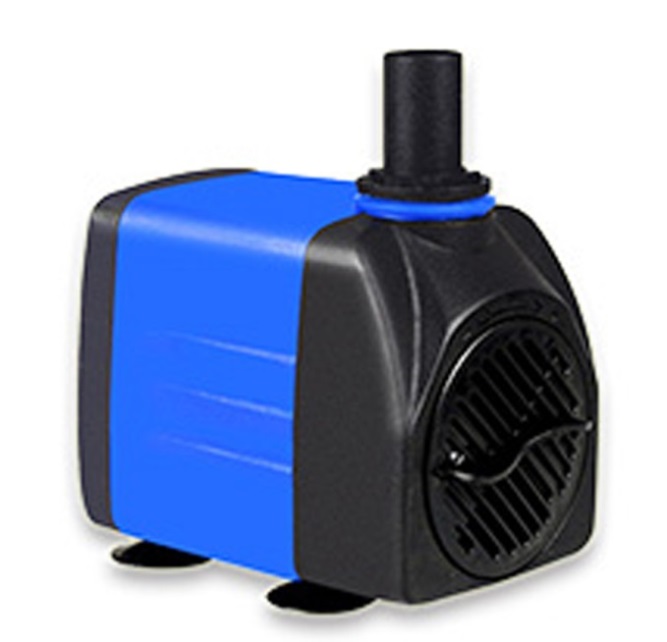


Figure S4. The photograph of submerged pump (Jiatong, JT160).

**Heat Exchanger Radiator (Dongyuan, 12S-8)**

The aluminum heat exchanger radiator (Dongyuan, 12S-8) is used for the heat-exchange. They have been extensively used as heat exchange radiator in the water-cooling system of computer. The copper radiator has a better heat dissipation capability but a higher price than the aluminum radiator. The size, shape and material of the radiator can be customized according to the heat dissipation demand of the system. A mini electric fan (5W, 12V) is mounted on the radiator to cool the radiator.


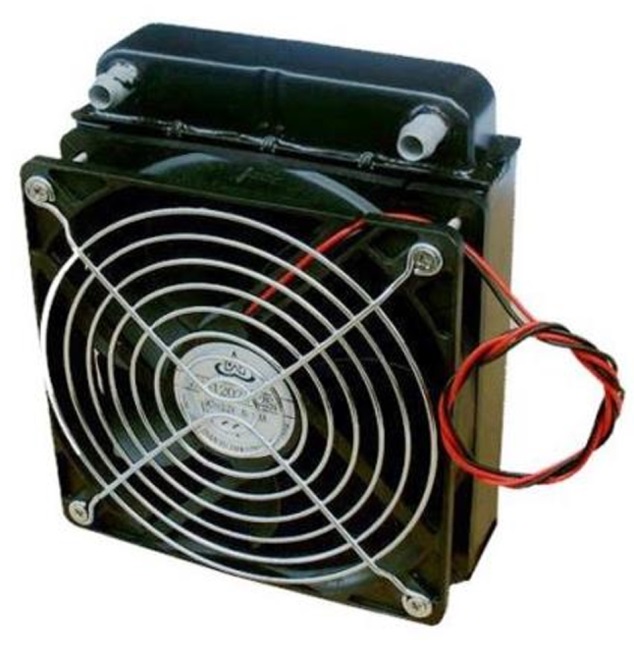


Figure S5. The photograph of heat exchanger radiator (Dongyuan, 12S-8).

Building Blocks of Self-made Multifunctional Voltage Regulator

**DC Voltage Power Capacity Indicator (XH-M241)**

XH-M241 is a DC voltage power capacity indicator percentage digital display module. The range of input voltage is 7 ~ 40V with the display precision of 0.1V. XH-M241 can display the input voltage and adjust the output voltage, using LM317 to fit the voltage for the device according to the input voltage.


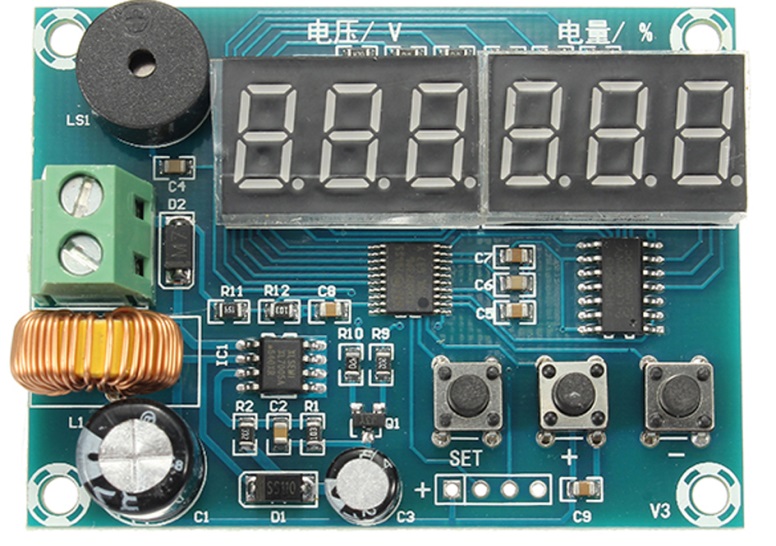


Figure S6. The photograph of DC voltage power capacity indicator percentage digital display module (XH-M241).

**DC step-down module (LM317)**

LM317 is a 3-terminal positive adjustable regulator. This monolithic integrated circuit is an adjustable 3-terminal positive-voltage regulator designed to supply more than 1.5A load current with an output voltage adjustable over a 1.2V to 37V range. It employs internal current limiting, thermal shutdown, and safe area compensation. LM317 can step down the input voltage of solar panel or acid-lead storage battery for the device.


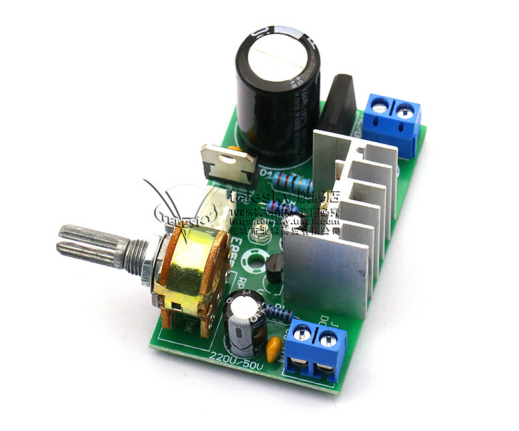

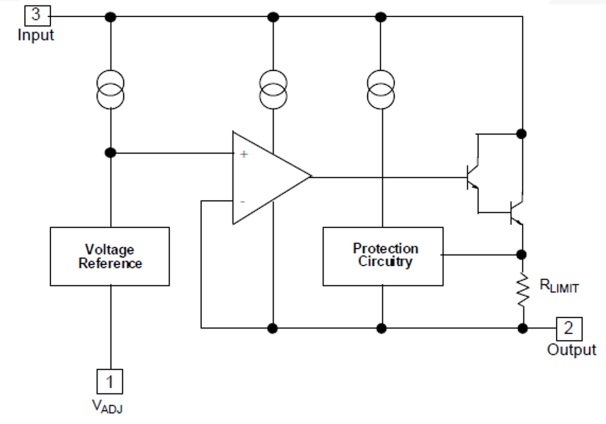


Figure S7. The photograph of LM317 (left) and its circuit diagram (right).

**DC boost module (LM2577)**

The LM2577 are monolithic integrated circuits that provide all of the power and control functions for step-up (boost), flyback, and forward converter switching regulators. Requiring a minimum number of external components, these regulators are cost effective, and simple to use. Listed in this data sheet are a family of standard inductors and flyback transformers designed to work with these switching regulators. Included on the chip is a 3.0A NPN switch and its associated protection circuitry, consisting of current and thermal limiting, and under voltage lockout. The output voltage versions of 9V and 12V are chosen for the multifunctional box.


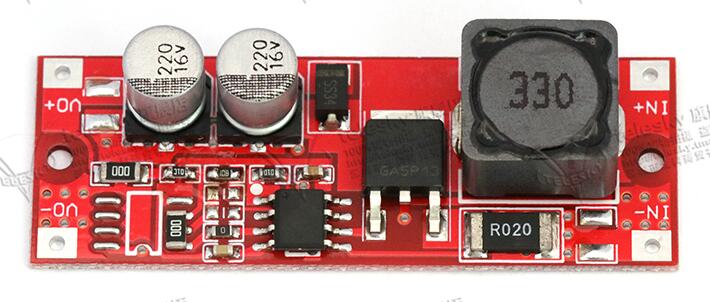

Figure S8. The photograph of LM2577 (top) and its circuit diagram(bottom).

PART II THE PRICE OF THOSE BUILDING BLOCK

| **Table S1. Main part list of proposed instrument with vendor and cost information** | | | | | |
| --- | --- | --- | --- | --- | --- |
| *Component* | *Vendor* | Part Number | Quantity | Cost (US, $) |  |
| Solar panel | www.tmall.com | 18V, 20W | 1 | 19.97 |  |
| Solar Charge Controller | www.tmall.com | BW-KZQ-10AK | 1 | 7.45 |  |
| Storage battery | www.tmall.com | GHGN-12V12AH | 1 | 20.29 |  |
| Submerged Pump | www.tmall.com | JiaTong, JT-160A | 1 | 3.80 |  |
| Heat Exchanger Radiator | www.aliexpress.com | AMZDEAL 120 | 1 | 13.74 |  |
| Aluminum bucket | www.tmall.com | YiLi 5L | 1 | 7.93 |  |
| DC boost module | www.tmall.com | LM2577 | 2 | 2.85 |  |
| DC step-down module | www.tmall.com | LM317 | 1 | 2.50 |  |
| Capacity indicator | www.tmall.com | XH-M241 | 1 | 3.25 |  |
|  |  | Total |  | 81.98 |  |

**Table S2 Main part list of proposed instrument with vendor and cost information**

| *Component* | *Vendor* | Part Number | Quantity | Cost (US, $) |
| --- | --- | --- | --- | --- |
| Solar panel | www.tmall.com | DJ-18V100WK | 1 | 63.08 |
| Solar Charge Controller | www.tmall.com | BW-KZQ-30AK | 1 | 16.32 |
| Storage battery | www.tmall.com | GHGN-12V100AH | 1 | 137.58 |
| Submerged Pump | www.tmall.com | JiaTong, JT-160A | 1 | 3.80 |
| Heat Exchanger Radiator | www.aliexpress.com | AMZDEAL 120 | 1 | 13.74 |
| Aluminum bucket | www.tmall.com | 5L | 1 | 7.93 |
| DC boost module | www.tmall.com | LM2577 | 2 | 2.85 |
| DC step-down module | www.tmall.com | LM317 | 1 | 2.50 |
| Capacity indicator | www.tmall.com | XH-M241 | 1 | 3.25 |
|  |  | Total |  | 251.05 |

The main part list, or bill of materials (BoM), is listed in Table S1 and Table S2, and the total cost for the demonstrating device is around $90, and the total cost for the practical device is about $250, it can provide cooling water for the lab for 5-10 years, which is still a cost-effective investment in the long run. The most important is that this device provides a very good model to cultivate the idea of water-saving and energy-saving to students.

Part III THEORETICAL CALCULATION

To achieve the goal of supplying electricity by solar panels and storage battery alternatively, the most important issue is to select solar panel with suitable power and a storage battery with enough capacity. The power of solar panels is determined by the daily power consumption of the device and local annual average sunshine time at standard irradiance.^1^ The power of our device is 10 W, and the nominal load voltage is 12 V and the working current is 0.833 A, so the daily energy consumption (Q_L_) was calculated according to the following formula (1):

Q_L_ = Load Voltage × Working Current × 24h (1)

=12 V × 0.833 A × 24 h = 240 Wh = 20 Ah

Total annual irradiation in Hubei province is 110 kcal/cm^2^ at the standard irradiance, and the averaged sunshine hours (H) can be calculated according to following formula (2):

H = (Total annual irradiation × 1.63 Wh/kcal) / (365 × 0.1 W/cm^2^) (2)

= (110 kcal/cm^2^ × 1.63 Wh/kcal) / (365 × 0.1 W/cm^2^) =4.91 h

The power of solar panel (P) was calculated according to formula (3):

P = (Q_L_ × V_L_ × Kop)/(Ioc × H × 12.5 V × C_C_) (3)

The daily power consumption of this device (Q_L_) is 20 Ah, the nominal load voltage (V_L_) is 12V, the best working current (Ioc) is 0.07 A/W, the discharge depth of storage battery (C_C_) is 80%, therefore the power of solar panel can be obtained.

P = (20 Ah × 12 V × 1.2)/(0.07 A/W × 4.91 h × 12.5 V × 0.80) = 83 W

Therefore, a solar panel with the output voltage of 12V and the power of 80 ~ 100W can fit with the demand of the device.

The capacity of storage battery (C) can be calculated according to formula (4):

C = Q_L_ × N_L_ ÷ C_Z_ (4)

The capacity of accumulator (C) is determined by the daily electricity consumption, and the largest time without sunshine (N_L_). The daily power consumption of this device is 240 Wh or 20 AH. The largest time without sunlight in Wuhan is taken the value of three days (72 h), the value of capacity coefficient (C_Z_) for acid-lead storage battery is 0.8.

The demanded capacity of storage battery is

C = 20Ah × 3 ÷ 0.8 = 75Ah.

Therefore, an acid-lead storage battery with the nominal voltage of 12V and capacity of 70 – 100Ah can fulfil the demand of this device in Hubei province (North latitude: 29°0′53″—33°6′47″).

ThE CHOICE OF THE MATERIAL OF WATER RESIVOR

| **Table S3. Thermal conductivity of different material**^2^ | | |  |
| --- | --- | --- | --- |
| *Material* | *Abbr.* | Therm. Conduc.  /(W m^–1^ K^–1^) | |
| Polypropylene | PP | 0.24 ± 0.002 | |
| Polymethyl acrylate | PMMA | 0.19 ± 0.005 | |
| Acrylonitrile Butadiene Styrene | ABS | 0.02 ~ 0.046 | |
| Tinplate | SPTE | 52.3 | |
| Copper | Cu | 385 | |
| Aluminum | Al | 205.7 | |
| Silver | Ag | 418 | |
| 304 Stainless Steel | SUS304 | 16.2 | |

The challenge of constructing a water recirculating cooling system is how to release the accumulated heat of the system to the environment rapidly. The heat exchange between the water bucket and the air also plays an important role besides the heat exchange radiator. It is well known that different bucket materials have different thermal conductivity and different cooling rates. Therefore, it is crucial to select suitable bucket material. A 5 L PP bucket (4 L water), a 5 L aluminum bucket (4 L water), and a 2 L tinplate bucket (2 L water) were selected to test their cooling rate. Temperature differences between the water in the bucket and the environment were recorded at an interval of 5 minutes. Cooling curves of different bucket material can be plotted using the cooling time as the X axis and temperature difference as the Y axis. As shown in Figure S9, all buckets have a high cooling rate in high temperature difference region, but aluminum buckets have the fast cooling rate in the low temperature difference region compared to other buckets. Therefore, an aluminum bucket is the best choice because this device will mainly work in a small temperature difference region. Moreover, aluminum has a better corrosion resistance than other common metals. Some other metals, such as copper and silver, have a better thermal conductivity than aluminum, but their cost is prohibitive. Therefore, the aluminum bucket is the best choice considering the material price, corrosion resistance, and thermal conductivity.


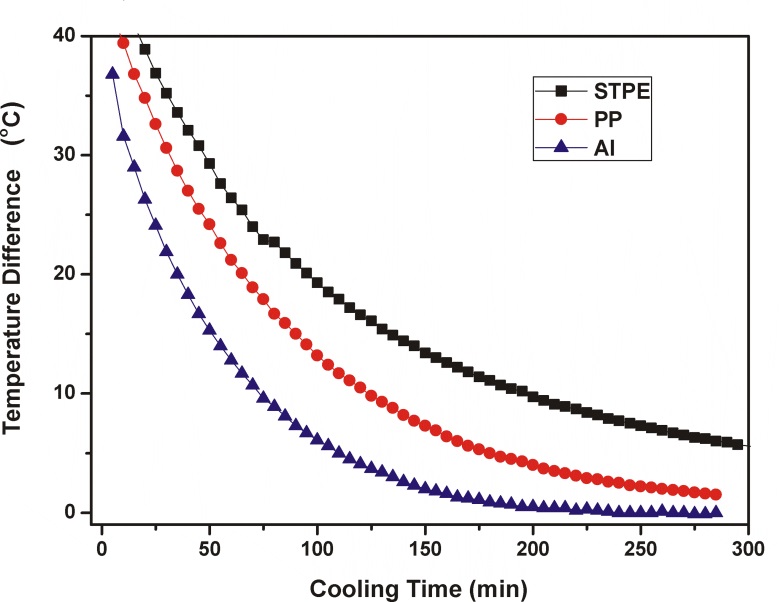


Figure S9 Cooling curve of different bucket materials (PP, STPE, Al).

| Table 2. Thermal data of selected solvents. [^3^](#_ENREF_13) | | | |  |
| --- | --- | --- | --- | --- |
| *Solvent* | *Water* | *Ethanol* | *Acetonitrile* | |
| Boiling Point | 100 | 80 | 80 | |
| Vaporization Heat(KJ/Kg) | 2256.9 | 838.07 | 767.35 | |
| Heat capacity (l, KJ/Kg·K) | 4.212 | 3.023 | 2.711 | |
| Heat capacity (g, KJ/Kg·K) | 1.872 | 1.600 | 1.388 | |

**Reference**

1. Li, Z., *Design, Installation and Application of Solar Photovoltaic Power Generation System*. Posts & Telecom Press: Beijing, **2012**.

2. Speight, J. G., *Lange's Handbook of Chemistry*. 16th ed.; McGRAW-HILL New York , Chicago, San Francisco, Lisbon, London, Madrid, Mexico City, Milan, New Delhi, San Juan Seoul, Singapore, Sydney, Toronto, **2005**.
